# X-change symposium: status and future of modern radiation oncology—from technology to biology

**DOI:** 10.1186/s13014-021-01758-w

**Published:** 2021-02-04

**Authors:** Stefanie Corradini, Maximilian Niyazi, Dirk Verellen, Vincenzo Valentini, Seán Walsh, Anca-L. Grosu, Kirsten Lauber, Amato Giaccia, Kristian Unger, Jürgen Debus, Bradley R. Pieters, Matthias Guckenberger, Suresh Senan, Wilfried Budach, Roland Rad, Julia Mayerle, Claus Belka

**Affiliations:** 1 Department of Radiation Oncology, University Hospital, LMU Munich, Marchioninistr. 15, 81377 Munich, Germany; 2 Department of Radiotherapy, Iridium Network, Faculty of Medicine and Health Sciences, University of Antwerp, Antwerp, Belgium; 3 Department of Radiation Oncology and Hematology, Fondazione Policlinico Universitario A.Gemelli IRCCS, Università Cattolica S. Cuore, Rome, Italy; 4 Oncoradiomics, Liège, Belgium; 5 Department of Radiation Oncology, Medical Center, Medical Faculty, University of Freiburg, Freiburg, Germany; 6 German Cancer Consortium (DKTK), Partner Site Freiburg, Freiburg, Germany; 7 Division of Radiation and Cancer Biology, Department of Radiation Oncology, Stanford University, Stanford, USA; 8 Integrative Biology Group, Helmholtz Zentrum Munich, Munich, Germany; 9 Department of Radiation Oncology, Heidelberg University Hospital, Heidelberg, Germany; 10 Department of Radiation Oncology, Amsterdam University Medical Centers, Location Academic Medical Center, University of Amsterdam, Amsterdam, The Netherlands; 11 Department of Radiation Oncology, University Hospital of Zurich, University of Zurich, Zurich, Switzerland; 12 Department of Radiation Oncology, Amsterdam University Medical Centers, Location VUmc, Amsterdam, The Netherlands; 13 Department of Radiation Oncology, Medical Faculty, Heinrich Heine University, Düsseldorf, Germany; 14 Center for Translational Cancer Research (TranslaTUM), TU Munich, Munich, Germany; 15 Department of Internal Medicine II, University Hospital, LMU, Munich, Germany

## Abstract

Future radiation oncology encompasses a broad spectrum of topics ranging from modern clinical trial design to treatment and imaging technology and biology. In more detail, the application of hybrid MRI devices in modern image-guided radiotherapy; the emerging field of radiomics; the role of molecular imaging using positron emission tomography and its integration into clinical routine; radiation biology with its future perspectives, the role of molecular signatures in prognostic modelling; as well as special treatment modalities such as brachytherapy or proton beam therapy are areas of rapid development. More clinically, radiation oncology will certainly find an important role in the management of oligometastasis. The treatment spectrum will also be widened by the rational integration of modern systemic targeted or immune therapies into multimodal treatment strategies. All these developments will require a concise rethinking of clinical trial design. This article reviews the current status and the potential developments in the field of radiation oncology as discussed by a panel of European and international experts sharing their vision during the “X-Change” symposium, held in July 2019 in Munich (Germany).

## Introduction

The symposium “XChange: Status and future of modern radiation oncology—from technology to biology” organized by the Department of Radiation Oncology, LMU Munich was held on July 26–27, 2019 in Munich, Germany. More than 150 radiation oncologists, medical physicists and RTTs attended this meeting. This review aims to summarize the important highlights of the meeting and to share the vision of the future of the field of radiation oncology. The manuscript contains the summaries of the individual contributions given by top scientists in the field of radiotherapy who report their personal views. Parts of the symposium were designed as competitive debate, where one panelist was assigned to a topic that was opposed by another panelist.

### Vision 2030

#### #Radiotherapy saves lives—*Maximilian Niyazi*

ESTRO has made a great job in drafting their new ESTRO vision 2030: “Radiation Oncology. Optimal Health for All, Together.” [1]. This is not just an arbitrary slogan—a very detailed description is attached, which defines the role of ESTRO in research, disseminating research, promoting the development of guidelines, advancing education, leading the international recognition of radiation oncology and much more. With this vision in mind, one could foresee a bright future—however, it makes sense to look back and define the true value of radiation oncology. What is the evidence that gives us certainty about the present and future role of radiation oncology? One such paper was published by Hanna et al. [2]—it reports on the population benefit of evidence-based radiotherapy, measuring 5-year local control (LC) and overall survival (OS) rates. From a methodological point of view, radiotherapy alone was compared to either no treatment or surgery alone; the added benefit of chemotherapy was assessed, as well as the comparison of chemoradiotherapy (CRT) over radiotherapy alone—all these analyses as meta-analytical approach with accompanying sensitivity analysis from a large country database. The results were overwhelmingly positive: 48% of all cancer patients have RT indications (34% curative), and 5y-LC benefit was 10.4% for all patients, while OS benefit was 2.4%. Overall, CRT adds 0.6% for LC and 0.3% for OS; the highest benefit was seen in head & neck cancer (H&N) with 32% LC and 16% OS benefit; and cervical cancer with 33% LC and 18% OS improvement [2].

However, what global impact will radiotherapy have in oncology? Lievens and colleagues published that one million lives could be saved by 2035. As cancer is composed by about 200 different cancer entities and 9 million cancer deaths have been observed in 2016, the projected number would be 14.5 million cancer deaths in 2025. Prevention could reduce this number by 40–50%, but much remains to be done in low-to-intermediate income countries [3]. However, saving these 1 million lives would require a global investment of 184 billion USD [3]. To confirm these epidemiological statistics: There are fantastic achievements of radiation oncology; curative and highly precise treatments of H&N, excessive improvements in stage III lung cancer (in combination with immunotherapy), SBRT as a new paradigm in oligometastatic disease—and these are just few examples, there is much more to come. Therefore, radiotherapy does save lives—but efforts must be made to provide a service to all who need it. Specifically, the access to radiotherapy is a mandatory precondition to save lives, and yet, for many cancer patients this is frequently a limiting factor in many low-to-intermediate income countries; therefore, political decision-making has to focus on technology and knowledge transfer.

### Present and future of MR linac—*Debate*

#### Critical appraisal “In 2030 MR linac will be limited to highly specialized centers”—*Dirk Verellen*

It is dangerous to make predictions about the future, and many great minds have fallen in that trap (google “predictions that were wrong” or look at https://list25.com/25-famous-predictions-that-were-proven-to-be-horribly-wrong/). The claim that MR-linacs will be limited to highly specialized centers could also be one of these mistakes. However, there are some valid arguments to at least claim that MR-linacs will not be the answer for mainstream radiation therapy practice. When reviewing the literature in the field of radiotherapy, one can observe a constant evolution of improvements in treatment delivery, with waves and hypes that come and go, some of which stay and become mainstream approaches. Some, even promising developments, unfortunately fade out to oblivion (remember the MM50 racetrack microtron [4] and recently the VERO-system for real-time tumor tracking and dynamic wave arc treatment [5]). Without being exclusive, one can observe a continuous improvement from kV-radiation, to ^60^Co-beams, MV-linacs, the introduction of CT and 3D dose calculation, improved dose calculation algorithms, conformal RT, MLC, IMRT, VMAT, IGRT, SBRT, etc., MR-linacs and proton therapy being the new kids on the block. As mentioned in the previous chapter, radiation therapy is a major and invaluable discipline in the fight against cancer, and improvements can only be encouraged. However, in face of today’s economic health care challenges and budget cuts, priorities have to be made and one has to ask the question: Will we invest in expensive tools for a small sub-group of patients or invest in tools that are mainstream accessible and improve RT quality for the majority of our patients? This and the next paragraph are the result of a point-counterpoint debate on the implementation of MR-linac in routine clinically practice, which is a nice example of the previous considerations.

Two main advantages of MR-linacs are often used to support its investment: the superior image quality compared to conventional kV-CBCT linacs and the potential for real-time adapted radiotherapy. Both arguments can be challenged. The combination of surface scanning (already a mainstream application in many centers [6–9]), daily CBCT and daily (accumulated) transmission dosimetry [10, 11] already allows for a truly adapted radiotherapy approach today. Surface scanning and gated/tracking techniques open the door to motion management for those limited cases that might benefit from these approaches (eg prostate SBRT, NSCLC and oligometastatic disease). New developments in machine and deep learning (ML/DL) [12] will soon enable markerless tracking and make complicated hybrid developments unnecessary. The argument on superior image quality is subject of an entirely different debate. Should we invest in integrating all the required imaging modalities into the treatment delivery machine, or is it more appropriate to apply state-of-the-art medical imaging using high-end imaging devices, and to merge this information at the treatment level of radiotherapy? In this way, a perfect synergy is created for individualized treatment, combining state-of-the-art images (both functional and anatomical) with robust tools for treatment monitoring (i.e. kV-CBCT and surface scanning). Again, current developments in ML and DL open the door to real-time image registration, automated segmentation and treatment planning, and biological conformal radiation therapy without the need to generate this information at the time of treatment. Low dose kV-CBCT with the aid of ML/DL provide an image quality that might even challenge MR-imaging for most IGRT-purposes [13]. MR-linacs can only provide limited information compared to the high-end medical imaging devices that are already in place in most oncology centers. Moreover, the technological challenges of combining MR- and linac-technology compromises the versatility of the treatment (e.g. large fields, non-coplanar treatment delivery and real-time surface scanning).

Finally, let us return to the argument of the cost to society and departments. The true cost of something is measured by what one has to give up to acquire it. Can we still offer individualized and truly adaptive radiotherapy in an environment where cuts in healthcare and an increased need for patient safety push towards more efficiency through standardization and automation? In other words, do we need state-of-the-art mainstream equipment for many patients or dedicated equipment for a few? Do we need standardized treatment or individualized treatment tailored to the patient? These questions are not the same and certainly not contradictory. It can be argued that standardization and automation of care pathways is the optimal way to achieve safety and quality within the complex workflow of radiotherapy, whilst at the same time offering a platform that allows personalized radiation therapy (including high precision and adaptive radiotherapy). For a patient the most important thing is not the availability of some high technology device, rather it is the ability of a team of physicians, physicists, dosimetrists and therapists to use the available technology with skill. It‘s the team, not the beam that makes a difference [14].

#### “In 2030 MR-Linac will replace CBCT”—*Vincenzo Valentini*

MR-Linac will certainly replace CBCT! But I have to disclose my bias: I love innovation, challenges and sustainability… and I have been using an MR-Linac since 2017.

**Fig. 1:**
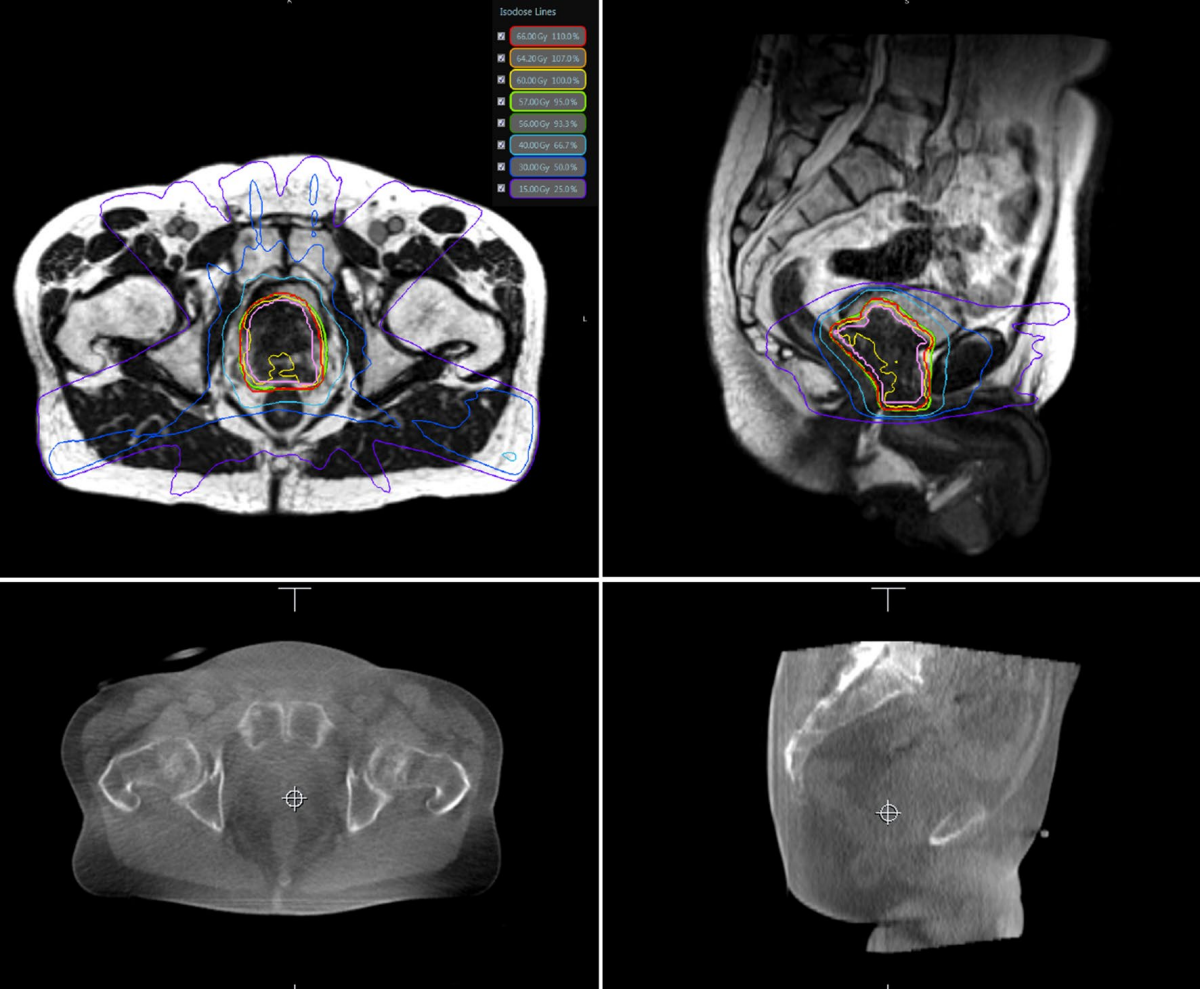
Exemplar MR-Linac based and Cone Beam CT based imaging of prostate cancer

The main advantages of MR-Linac are based on the way it solves image-guided radiotherapy (IGRT) and the possibilities that this technology opens up for radiation oncology practice [15–18]. There are three main evident advantages of on board MR-Linac compared to cone beam computed tomography (CBCT) IGRT: better imaging for (1) daily positioning of the patient; (2) monitoring during treatment delivery; and (3) online adapting the treatment plan to the daily anatomy. Regarding point 1, MRI has better soft tissue contrast and no additional radiation exposure compared to CBCT, which allows a direct visualization of the target. An example is given in Fig. 1. With respect to the second aspect, depending on clinical needs, it is possible to monitor the moving target volume directly or indirectly [19], or the organs at risk online and in real time—throughout the entire treatment fraction [20]. In order to be able to deal with all movements in daily treatment, an analysis to identify movement variations such as baseline drifts and shifts has identified a threshold for significant geometrical uncertainties that allows MRI-based real-time monitoring or an active gating approach for all lesions affected by respiratory movements above this threshold [21].

As far as online plan adaptation by MR-Linac is concerned, this approach is currently superior to a standard IGRT approach based on many advantages: imaging prior to the start of treatment and on-table re-contouring and re-planning allows for a correction of interfraction day-to-day variations by high resolution images (e.g. prostate, pancreatic cancer). These advantages close the gap of the many uncertainties of offline adaptation [22].

Moreover, MR-Linac supports further advantages of online imaging: outcome prediction by radiomics, toxicity monitoring by soft tissues imaging and dose painting by diffusion-weighted image sequences [23, 24]. With regard to the sustainability of this technology, as observed in previous implementations of new technologies in medicine, the cost of MR-Linac will decrease over time and the ability to identify suitable patients will support and accelerate its implementation.

Thanks to the described technological advances, MR-Linac will play an innovative role in the always more contemporary frame of a fully personalized care, adapting radiotherapy treatments to the single patient needs and successfully moving from standard irradiation techniques to a tailored treatment approach.

Rather than scattering patients towards standard high volume, lower cost but poorly personalized IGRT approaches, MR-Linac will pave the way to the introduction of a new concept of treatment personalization and comprehensive oncological care aiming to summarize high quality radiotherapy delivery, advanced imaging information (multimodal, functional imaging and radiomics) and effective motion management.

So,will MR-Linac replace CBCT? For sure, it will. But not for all tumours, not for all patients, and not for all treatments.

### You can only treat what you see

#### Buzzword radiomics: the bridge between medical imaging and personalized medicine—*Seán Walsh*

Radiomics is defined as high-throughput machine learned quantitative image features from standard-of‑care medical imaging that enables actionable insight to be extracted and applied within clinical-decision support systems to improve diagnostic, prognostic, and predictive accuracy. In oncology, this refers to the comprehensive quantification of tumor phenotypes and is a promising field of scientific investigation with a large amount of activity in recent times [25].

Radiomics can offer a potential partial solution to the grand challenges of oncology [26, 27], as one of the pain points is the difficulty in proving the effectiveness of treatments. Currently, classical clinical endpoints are not sensitive enough, which results in many compounds ultimately failing in phase III studies, because they have a weak correlation to phase III endpoints [28]; in addition, they provide only a crude measurement of the target, and require large patient populations, and long follow-up times. In this context, radiomics has the capability to become a next generation clinical endpoint, as it is a non-invasive way of having an instantaneously 3D (or 4D) local and global quantification of the tumor response.

Imaging is ubiquitous in medicine [29] as it offers non-invasive, 3D and easy to repeat measurements of the patient and disease. This is vital in the context of oncology, as most cancers are spatially and temporally heterogeneous [30]. The three primary options in imaging to tackle these issues are (1) new hardware: expensive, needs staff training, and maintenance, (2) new tracer: expensive, challenging to logistics, and single use, (3) new software: affordable, automated, and with multiple uses. These properties are driving the economic dynamics and reality of imaging development [31].

Therefore, the central hypothesis is that radiomics will disrupt current interpretative-subjective imaging description by providing definitive-objective imaging characterization [32]. This is inevitable when machines are compared to humans [33–36] for specific tasks. The workflow [37] begins with the acquisition of images/data (including QA and curation if necessary), the identification of regions of interest (automatically or manually), the pre-processing, extraction of features (handcrafted or deep), and post-processing of features, and machine learning (training of application) is then performed, culminating in a link to clinically actionable insight (diagnosis [38], prognosis [39], theragnosis [40], or follow-up [41]).

A major criticism of AI in general is the lack of (perceived) transparency, typically referred to as a ‘black box’ objection [42–45]. One aspect of this is purely AI mathematics [46], the other aspect is linking this elucidation to our current understanding in terms of clinical cancer biology [47]. This is now a rich field of research [48] and is beginning to produce tangible and significant results (genomics [49], histology [50], molecular biology [51], etc.). This research will help to define the biological basis of AI/radiomics [52] and facilitate successful crossing of the translational gaps [53] towards investigational and ultimately routine clinical practice.

Currently, no study has demonstrated clinical level 1evidence (i.e. prospective study [54]) for any radiomics signature. Until this hurdle is crossed, the implication for the field is that it is still in the experimental retrospective research stage of development. However, there are important methodological approaches (e.g., the TRIPOD statement [55] and the Radiomics Quality Score [25]), which are assisting in the selection of candidate radiomics signatures for prospective validation.

Taken together, radiomics is an emerging field that translates medical images into quantitative data to provide biological information and enables clinically actionable insights (diagnosis, prognosis, theragnosis, or follow-up).

#### Quo vadis PET-guided radiotherapy?*—Anca-Ligia Grosu*

The first rationale for using positron emission tomography (PET) for radiation treatment planning is its high sensitivity and specificity for tumor tissue [56–63]. This was observed in histological studies of many malignant diseases comparing PET to traditional radiological examinations such as CT and MRI. Considering that target volume delineation is a `condition sine qua non´ for high-precision radiotherapy, the introduction of FDG-PET in lung cancer [64], amino acids –PET in brain tumors [65, 66] or PSMA-PET/CT in prostate cancer [67, 68] significantly improved the accuracy of treatment delivery and consequently the clinical outcome [64, 65, 68, 69].

The second rationale for the integration of PET into radiotherapy planning is its ability to visualize molecular-biological pathways, which can subsequently be targeted by irradiation [70]. The imaging of tumor hypoxia, proliferation, angiogenesis, apoptosis etc. enables to recognize the enormous heterogenesis of malignant tissue, and accordingly to define subvolumes in the tumor, the so-called biological target volume, which needs to be targeted using different irradiation doses or fractionations. This approach, which is closely related to the technique of intensity modulated radiotherapy (IMRT) and IGRT gave rise to the concept of dose painting [70, 71]. For example, visualization of hypoxic subvolumes [72] and quantification of tumor hypoxia under chemoradiotherapy [73–77] lead to the concept of individual hypoxia-PET-based dose escalation in patients with advanced H&N cancer treated with primary definitive chemoradiotherapy. Moreover, the visualization of tumor receptors (for example stem cells receptors in malignant gliomas, [78]), gene expression, proteins, immunological response [79] etc., will allow a personalized irradiation treatment based of the molecular characteristics of tumor and normal tissue.

Artificial intelligence (AI) will significantly improve the understanding and use of imaging for planning and monitoring radiation treatment. AI will help to correlate the physical properties of the images with the biological features of the tissue and the clinical outcome: Imaging are not only pictures, they are data [32]. Radiomics features, extracted from the PSMA-PET data, for example, allow the detection of the Gleason score in prostate cancer in vivo [80]. New radiation treatment planning algorithms will take into account the biological properties of tumors and healthy tissue, as registered in the imaging. The probability of tumor control (TCP) can be balanced with the probability of side effects (normal tissue complication probability, NTCP) to find the optimal, personalized dose and fractionation. New devices such as PET/MRI, MR/LINAC, PET/LINAC will show the morphology and function of the tissue during the treatment and will allow to adjust the dose and fractionation on the fly. In summary, PET will play a significant role in radiation oncology in the future to achieve the essential goals of modern oncology: precision, personalization and individualization.

### Radiation biology on the move

#### Advances in radiation biology—*Claus Belka*

Besides the pronounced technological advance in the area, future radiation-based treatment strategies will be strongly influenced by biological research. Several areas of progress can be delineated: Molecular genetics of cancer pathogenesis, cell death mechanisms, DNA damage detection and repair, immune biology, rationally designed biologically combined modalities, marker-based stratification, radiation biology of altered fractionation i.e. FLASH and targeted interference with signaling pathways associated with side effects.

During the X-Change meeting 2019 Kirsten Lauber (Munich), Roland Rad (Munich), Amato Giaccia (Oxford) introduced and discussed recent developments in the aforementioned fields.

Understanding the basic mechanisms of specific cell death and the subsequent steps of immune presentation will allow for a specific interference with immune signaling and will ultimately boost the efficiency of radiation treatments. Multiple lines of evidence prove that radiation exerts a complex pattern of cell death events each being associated with multiple and diverging immune reactions [81, 82]. For example, after irradiation of breast cancer cells a different pattern of cell death and immune cell recognition is evident depending on the underlying genetic pattern of breast cancer [83]. Astonishingly, the specific immune response may also be subject to secondary interferences. In this regard it could be shown that HSP90 inhibitors strongly increase immune priming of tumor cells after irradiation [84].

Currently, a multitude of different approaches of combining “immune therapy” with irradiation is in preclinical testing, in early clinical testing or has already entered early clinical routine. The most prominent approach is the targeted interference with negative immune regulation by either CTLA-4 or—even more—PD1/PD-L1 system.

In case of lung cancer adjuvant application of durvalumab shortly after radio-chemotherapy for locally advanced non-small cell cancer has been shown to increase survival [85, 86]—although the parallel application of checkpoint inhibitors with concomitant radio-chemotherapy is feasible the clinical value is not yet fully defined [87].

Closely interwoven with the immune system several other target structures may be approached in order to specifically increase radiation or drug mediated killing of tumor cells. Hypoxia and associated metabolic pathways [88–90] may be also of value as well as highly defined signaling cascades like the GAS6/AXL pathway [91]. Thus, searches for specific survival-signaling pathways and also cell death pathways may open new horizons for synthetic lethality approaches in combination with ionizing radiation.

In close proximity to biological approaches dealing with tumor hypoxia, a new more physics related technology may help to overcome the adverse effects of hypoxia. FLASH-irradiation has been shown to more effectively target tumor cell when compared to non-malignant counterparts [92–94]. The underlying background is currently poorly understood—in case of lung irradiation the upregulation of fibrosis related genes and senescence induction is reduced. The effect of FLASH-irradiation seems to be critically related to the oxygen level being present [95]. In this regard a pure physical phenomenon directly translates into biological effects.

Finally, deciphering the molecular pathways leading to radiation induced toxicity will ultimately open new doors for molecular approaches heading for an increased therapeutic gain [96].

The need for better predictors and prognosticators is evident—even in 2020, most treatment concept lack a rational for adequate individualization of the underlying indication to treat, the respective combination partner and the radiation dose needed. Several—mostly omics-based approaches are available for glioblastoma [97], head and neck cancer [98–100] prostate cancer [101] and even for the prediction of lymph-node metastasis in cervical cancer [102]. Nevertheless, none of these have been validated for clinical decision making with the underlying problems nicely described in a recent commentary [103].

Finally, all approaches heading for a fundamental understanding of the genetic basis of tumor development are crucial for any of the aforementioned tasks [104]. Using pancreatic cancer as example, it has become clear that drivers like Ras in relation to gene dosage and evolutionary pattern determine the ultimate phenotype of the malignancy [105]. Without these fundamental studies, any approaches specifically targeting a given disturbance would not have been possible [106, 107].

#### Prognostic modelling in radiation oncology—*Kristian Unger*

The selection of therapeutic treatments in oncology is based on diagnostic decisions that mostly rely on the pathology of the tumors, molecular markers and clinical performance scores. However, for many cancer entities, such as glioblastoma (GBM) or locally advanced head and neck squamous cell carcinoma (HNSCC) the resulting therapeutic strata still are heterogeneous with regard to clinical outcome in terms of survival and recurrence. For this reason, the search for molecular signatures that enable the definition of prognostic substrata and an individual assignment to these is a substantial part of computational personalized medicine approaches. Personalized medicine aims to offer cancer patients the most individualized diagnosis and treatment possible. Molecular prediction rules are the prerequisite for this.

**Fig. 2:**
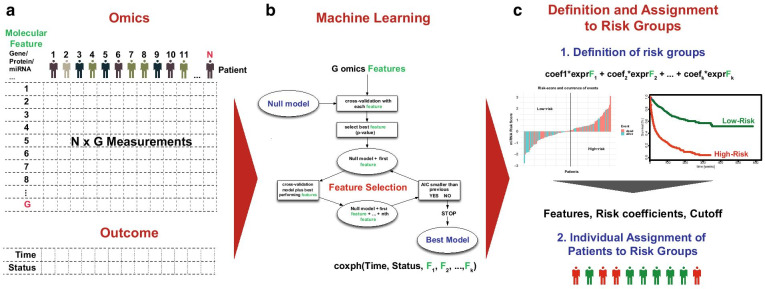
Machine learning in prognostic modelling using omics data. **a** Omics data consisting of N (number of patients) times G (number of molecular features) measurements in combination with time-to-event data are the basis in prognostic machine learning. Features can be measurements from any molecular layer such as the genome, transcriptome, proteome, post-transcriptome or epigenome. **b** Machine learning selects the molecular features to be added to a Cox pH model in such a way that the time-to-event data are optimally explained, while overfitting of data must be prevented. Here, exemplarily iterative forward-selection is shown. **c** The best model is used to calculate risk scores for all patients that allow, in combination with a cutoff, assignment of patients to high- or low-risk groups with regard to the chosen time-to-event endpoint (e.g. overall survival). The model coefficients for each molecular feature of the model, in combination with the defined risk score cutoff, allow the typing of new patients assigned to risk groups and thus the prediction of the individual risk for the endpoint of interest

Computational prediction modelling uses two elements for the generation of prognostic signatures: omics data and clinical outcome data (see Fig. 2). The clinical outcome, which is technically time-to-event data, is mathematically described using the Cox proportional-hazard (Cox-PH) model. To create a molecular prognostic signature, omics features such as genes, miRNAs, proteins or metabolites are added to the basic Cox model as covariates to achieve a superior fitting of the data. The process of feature selection is carried out using machine learning, which is part of artificial intelligence and is used to find the best selection i.e. the signature of molecular features to be added to the cox model as covariates for a significantly improved model fit while being of low-complexity. The latter increases the chance that the signature is transferable to other data sets and data from individual patients.

A large number of molecular prognostic signatures have been published in recent years, however, only a few made it into clinical practice, such as Oncotype DX or Mammaprint in breast cancer. Possible reasons for the low success rate here are small, non-representative discovery cohorts, flawed study designs and inappropriate choices of bioinformatics approaches. An important decision point is the selection of discovery and external validation cohorts, while there is a discussion ongoing whether multicentric or monocentric cohorts should be used and which combination increases the chance of finding generalizable molecular signatures. Another challenge with molecular signatures is the lack of suggestions on how new prognostic factors can be integrated into the framework of existing robust clinical markers or other existing molecular signatures from other molecular levels. For this reason, an important research topic in this field is the conception of multilevel prediction approaches that allow the simultaneous generation of signatures at several molecular levels, clinical markers and existing molecular signatures.

### The bigger the better?—*Debate*

#### Brachytherapy and proton beam therapy, hand-in-hand for optimal care.—*Bradley R. Pieters*

Proton therapy (PT) was introduced as an alternative technique to conventional photon therapy (PhT) [108]. Both irradiation techniques are in fact external beam radiation techniques. The main advantage of PT is that due to beam characteristics, PT is better able to deposit the ionizing energy within the target volume and avoid healthy organs and normal structures nearby. The focused deposition of energy with PT is caused by the phenomenon that protons release their energy at the end of their travel track through tissue (Bragg-peak). Due to the limited facilities in PT centers and the high treatment costs, PT is still offered mainly for indications where it is expected to be beneficial compared to PhT. Such standard indications are, for example, skull base tumors or pediatric tumors [109]. For several of late indications, predictions effects can be used by comparing PhT plans to PT plans in the absence of randomized studies to decide on the preferred treatment, the so-called model-based approach [110, 111].

The introduction of PT has not only an impact on the use of conventional PhT, but also on other radiation treatments such as brachytherapy (BT). With BT, radioactive sources are placed in or near the target volume to deliver radioactive energy directly to the target volume without the need to pass through the body. Obvious similarities between PT and BT are the steep dose gradient of dose distribution, which makes both modalities suitable for conformal radiation treatments and allows reducing low dose exposure.

However, there are more differences between the two modalities than similarities (see Table 1). The major disadvantage of PT compared to BT is the existing uncertainty in dose distribution caused by patient positioning and variation in density of the planning CT [112]. Another disadvantage for PT is the costs involved in the treatment [113]. On the other hand, a main disadvantage of BT is that not all tumor localizations are accessible for implantation of the sources, either directly or via guiding applicators. BT is an invasive procedure in most cases, therefore, the traumatic injury caused by the implantation must also be taken into account. Target volume size is another aspect to consider in the comparison between PT and BT. A very large target volume, usually an elective area, cannot be treated by brachytherapy, while size is usually not a problem for PT. For example, it is impossible to treat an entire pelvic area with brachytherapy, while this is not a problem for any kind of external beam therapy, including PT.

**Table 1:** Differences between proton beam radiotherapy and brachytherapy

Proton beam therapy	Brachytherapy
External beam radiotherapy	Internal irradiation
Very small to large size PTVs	Very small to intermediate size PTVs
Commonly homogeneous dose distribution	Heterogeneous dose distribution
Almost all locations can be treated	Limited in locations to treat
Beam specific margins to account for dose distribution uncertainties	Limited or no PTV margins

In-silico planning comparisons can provide some insight in differences between PT and BT. Georg et al. compared BT plans to intensity modulated PT plans for cervix cancer treatment [114]. In this plan comparison it was found that it is possible to achieve comparable dose to the high-risk planning target volume (HR-PTV) with PT as with BT, although the dose to the gross tumor volume (GTV) was lower with PT. Another difference found in this comparison was the mean 60 Gy volume. This volume, which is related to the probability of late toxicity, was 1.5 times larger with PT compared to BT. A similar study from the same group was done for prostate cancer treatment [115]. Very limited data are available on the clinical comparison between BT and PT. Some comparative clinical studies on uveal melanoma are published with a disparity in result [116–118].

Despite the lack of comparative studies between BT and PT, it is clear that for certain indications such as cervical and prostate cancer, the addition of BT is expected to improve tumor control [119–125]. Although these studies were comparisons between PhT and BT, it is not expected that PT will result in better tumor control than PhT. The improved tumor control can only be explained by the characteristics of BT, which delivers very high doses within the implant [126]. The advantage of PT over PhT is in the reduction of toxicity, although also with PT toxicity is reported [127]. When addressing the benefits of BT and PT, it is merely a matter of exploring the added value of one treatment over the other. In certain indications where brachytherapy is not possible, PT may be useful because of the possibly lower likelihood of toxicity. On the other hand, if a dose-escalation is desired, BT is the most designated technique to achieve this.

### Radiation Oncology and multimodal treatments

#### Oligometastasis and local ablation in the era of systemic, targeted and immunotherapies.—*Matthias Guckenberger*

After the first description of oligometastatic disease (OMD) as a distinct cancer stage between locally confined and systemically metastasized disease by Hellman and Weichselbaum in 1995 [128], this concept is today supported by a growing number of high-quality trials. Three randomized trials reported an improvement in progression-free survival (PFS) [129] or overall survival (OS) [130, 131] by the addition of local metastases-directed therapy to standard-of-care systemic therapy. Palma et al. described an OS benefit of metastases-directed stereotactic body radiotherapy (SBRT) in a tumor-agnostic trial [132]. Ost et al. compared metastasis-directed SBRT with surveillance in oligorecurrent prostate cancer and reported improved androgen deprivation therapy free survival [133]. In metastatic prostate cancer, local radiotherapy of the prostate improved OS in the situation of low metastatic burden compared to androgen deprivation therapy only [134]. Based on these positive studies, the concept of radical local treatment with curative intent in OMD has been rapidly implemented in the oncology community. Especially stereotactic radiotherapy is recognized as favorable local treatment modality [135], which achieves high rates of local metastases control with usually low toxicity, is delivered in few treatment sessions, allows simultaneous treatment of several targets at distant sites, and can be well integrated into multimodality treatment concepts [136, 137].

Despite these advances, many challenges remain and require well-designed clinical trials and translational research activities:

Limited progress has been made in understanding and defining OMD based on biology, i.e. in recognizing patients with truly limited metastatic capacity, based on OMD-specific biomarkers [138–142]: external or independent validation has been either unsuccessful or is still lacking.

The current lack of biomarkers has made imaging the most relevant diagnostic modality for defining OMD [143]. However, limited numbers of metastases on imaging may represent very different clinical scenarios, which are associated with different prognosis and may require different treatment strategies. This indicates the need for a comprehensive system for OMD characterization and classification [144].

After radical local treatment, the majority of the patients will ultimately develop distant disease progression [130, 134, 145, 146]. This indicates the need for more effective systemic therapies integrated into multimodality treatment concepts. Especially the combination of stereotactic radiotherapy with immune checkpoint inhibition appears promising due to the immune-enhancing effect of radiotherapy.

Timing and sequencing of staging, local and systemic treatment have become challenging in OMD due to the high variability in the clinical presentation of OMD. Additionally, the choices of local and systemic treatment modalities are highly relevant due to their potential interactions.

It is obvious that different study designs are required to address all relevant questions described above: prospective interventional trials with traditional and modern designs, such as basket or umbrella trials, to answer proof-of-principle questions, as well as registry trials to assess real-world data in a timely manner.

#### Local control versus distant control in lung cancer: adequate integration of radiotherapy—*Suresh Senan*

Major advances have been made in the systemic therapy of lung cancer, which in some cases, has led to a reassessment of indications for radiotherapy, timing and also the preferred dose-fractionation schemes. These developments can be illustrated using the examples of the new paradigm in inoperable stage III NSCLC, and oligometastatic lung cancer.

In patients with inoperable stage III NSCLC, the standard of care has now evolved to become concurrent chemoradiotherapy to a dose of 60 Gy, followed by administration of 12 months of immune checkpoint blockade using durvalumab, an anti PD-L1 antibody [85]. The PACIFIC trial reported a statistically significant and clinically meaningful improvement in both, overall survival and progression-free survivals versus placebo. In addition, improved intrathoracic disease control in the durvalumab arm provided evidence for the enhancement of radiation-induced local effects.

The clinical findings of PACIFIC were not entirely consistent with findings from animal studies of optimal fractionated radiotherapy with immune checkpoint blockade. Using 2 murine models and PD-1 blockade, which started 7 days after the end of radiotherapy administered with once-daily fractions of 2 Gy, it was shown that the delayed sequence of checkpoint inhibition was ineffective [147]. This animal model suggested that the exhaustion and atrophy of tumor-reactive T-cell responses may occur rapidly after radiotherapy unless the PD-1/PD-L1 axis is blocked. Ongoing studies in stage III NSCLC are exploring the efficacy of chemoradiotherapy concurrently with immune-checkpoint blockade, as well as sequencing multiple immune checkpoint blockade.

The progress made in metastatic NSCLC has been using both, systemic therapies and SABR. Two small trials in patients presenting with synchronous oligometastatic NSCLC revealed survival improvements with the addition of locally ablative therapy [130, 145], and a larger trial is underway to validate these findings (NCT03137771). In patients with a controlled primary tumor, the SABR-COMET trial reported a statistically significant improvement in progression-free survivals with the addition of SABR to 5 or fewer metastases, versus only standard of care [132]. With an extended follow-up to a median of 51 months, the impact of SABR on 5-year overall survival OS was larger in magnitude (42.3% vs. 17.7%, *P* = 0.006) than in the initial analysis and durable over time [148]. The findings of this landmark study have stimulated further studies in oligometastatic disease, including the phase III trial SABR-COMET-3 (NCT03862911) and SABR-COMET-10 (NCT03721341) trials.

### Theoretical considerations on multimodal treatments in 2030—*Wilfried Budach*

**Fig. 3:**
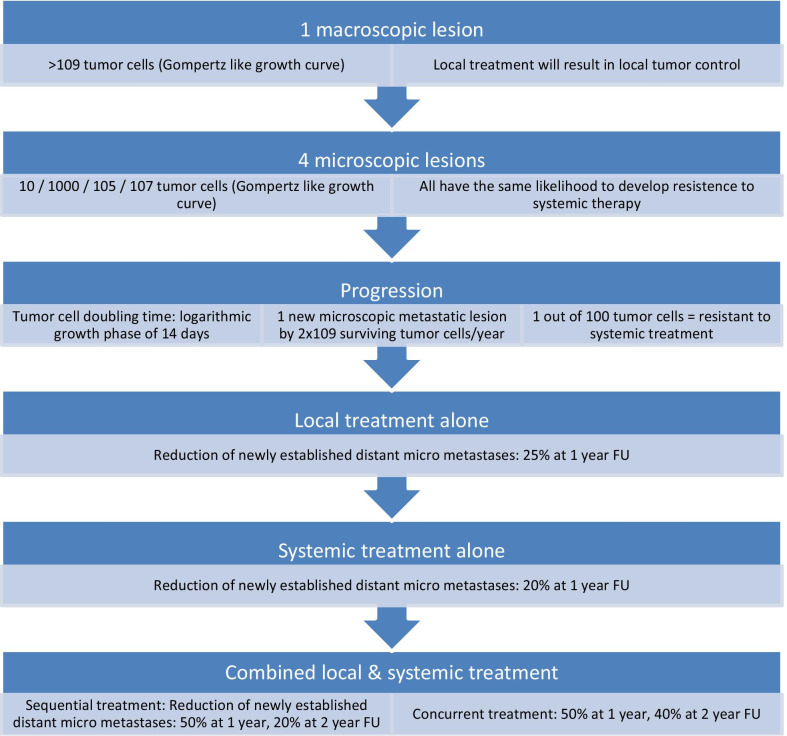
In silico modelling of metastatic spread and the impact of local or systemic therapy. FU: follow-up

Local treatments such as surgery and radiotherapy are the only treatment modalities that are able to cure cancers when the risk of cancers cells outside the treated tissue is low. A cure of cancer with systemic treatment alone is possible, if the respective drugs eliminate all tumor cells, which typically requires > 10 orders of magnitude of tumor cell killing. For most solid cancers, both prerequisites are typically not met, suggesting that combined modality treatments should lead to a better clinical outcome, as has been shown for several cancers, especially in locally advanced disease (see Fig. 3).

For metastatic disease of solid cancers, the benefits of local treatments are less obvious and still a matter of debate. A small number of randomized trials in the oligometastatic (≤ 5 lesions) setting suggest that local treatments improve PFS and overall survival [130–134, 145, 149]. However, the optimal timing of these combined treatments is not yet known.

Immune checkpoint inhibitors (ICPB) have already revolutionized the treatment of several cancers in metastatic disease and in locally advanced NSCLC. Experimental data suggest that a tumor primarily resistant to ICPB can be reverted back into a sensitive tumor by adding concurrent radiotherapy [150, 151]. It is assumed that the immunogenic tumor cell death after radiotherapy is the mechanism behind these observations. The optimal radiation dose and fraction size to achieve this effect is still controversial in view of conflicting data. Many experts believe that fractions sizes of 4–8 Gy could be optimal. Recently, it has been shown that functionally intact regional lymph nodes are important to establish this radiation induced immune priming and that in takes approximately 7–14 days after radiotherapy until the maximal immune effect has been established [152–154]. Nevertheless, currently recruiting clinical trials on the combination of radiotherapy and ICPB in locally advanced disease largely do not take these findings into consideration.

**Fig. 4:**
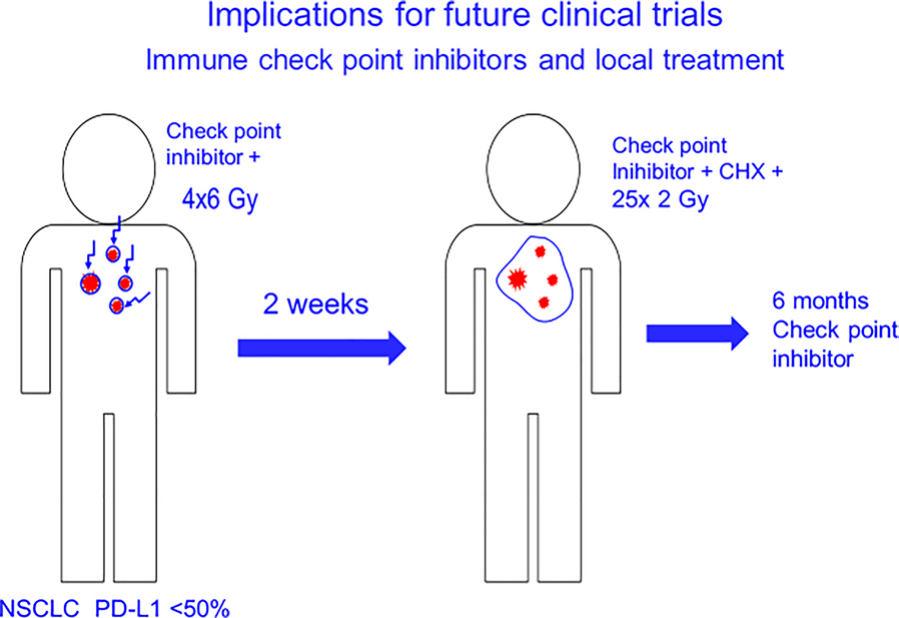
Theoretical considerations on multimodal treatments in 2030

Based on the current knowledge, the experimental arm of a trial testing ICPB in combination with radiotherapy in locally advanced solid tumors could be designed as follows (see Fig. 4):

1. Limit inclusion to tumors that are unlikely to respond very well to ICBP alone (CPS < 20%-50%)
2. Start ICPB 1–3 days before radiotherapy
3. Irradiate all macroscopic tumor with little margin, with 4–5 × 4–6 Gy within 1 week. Try to minimize radiation to regional lymph nodes without tumor involvement.
4. Re-biopsy / restaging approximately one week after the last radiotherapy
5. Start subsequent definite local treatment (surgery ± adjuvant radiotherapy ± chemotherapy or concurrent chemoradiation (no boost RT))
6. If the biopsy 1 week after initiation of RT + ICPB indicates an immune response, continue ICBP therapy for 6–12 months.
7. Consider to make the choice of the definite local treatment depending on the immune response to induction ICPB + RT: Definite RT (no CHX) in case of good response, surgery + adjuvant RT or RT-CHX in case of no/minor response (concurrent RT-CHX, if surgery is not possible with reasonable toxicity)

### Vision and reality

#### Considerations for clinical trial designs—*Julia Mayerle*

"If it were not for the great variability among individuals, medicine might as well be a science and not an art." -Sir William Osler (1892) [155]

Historically, clinical decision-making has been dictated by the clinician's experience, which has frequently been biased and constrained by limitations in available scientific knowledge. To overcome these limitations, clinical trials were introduced in the eighteenth century. A well-known example of an early case–control study performed by James Lind, was the introduction of citrus fruits to prevent scurvy in the Royal Navy. However, it took another 200 years until the advent of randomized controlled trials (RCT) in the 1940s to reduce bias through randomization and prospective data collection [156].

The focus of traditional clinical trials was identified in the 1979 Belmont Report [157]—the bible of clinical research ethics- which emphasized that clinical research is distinct from clinical practice. Similarly, the regulatory authorities are required to focus on safety and efficacy when evaluating medical products [158]. The standards for determining safety and efficacy are the same for all diseases and conditions, regardless of the disease prevalence. A fundamental principle in traditional trial design is to understand and control the false-positive rate. Compliance with this principle requires large trials and very large sample sizes. Unfortunately, traditional RCTs do not take into account the many facets caused by biological variations [159]. This is even more evident in complex diseases such as cancer or benign diseases, which are accompanied by many underlying genetic predispositions or environmental factors. It is more challenging to develop therapies in rare disorders, such as individual cancer subtypes or their subsets as they never fit well into the traditional ways of trial planning [160].

The "precision medicine trials" are particularly challenging. There is a growing interest in conducting mechanism-based trials where eligibility is based on molecular targets rather than traditional disease based definitions. One approach to conducting such trials would be to establish a master protocol combining umbrella trials (to investigate multiple targeted therapies in the context of a single disease), basket trials (to investigate a single targeted therapy in the context of multiple diseases or disease subtypes), and platform trials (to investigate multiple targeted therapies in the context of a single disease in a perpetual manner, with therapies allowed to enter or leave the platform based on a decision algorithm) [161]. All of them represent a collection of trials or substudies that have important design components and operational aspects in common. Such adaptive trial designs, following a master protocol, offer a way forward for heterogeneous and low-incidence diseases with high medical needs, such as cancer. It should be noted that even a master protocol and an adaptive trial design will require a valid endpoint; if no cure can be achieved but palliation, aspects other than progression free survival or overall survival might get into focus [162].

## Data Availability

Not applicable.

## Abbreviations

AI: Artificial intelligence
BT: Brachytherapy
CBCT: Cone beam computed tomography
CHX: Chemotherapy
Cox-PH: Cox proportional-hazard
CRT: Chemoradiotherapy
GBM: Glioblastoma
GTV: Gross tumor volume
H&N: Head & neck cancer
HNSCC: Head and neck squamous cell carcinoma
HR-PTV: High-risk planning target volume
ICPB: Immune checkpoint inhibitors
IGRT: Image-guided radiation therapy
IMRT: Intensity modulated radiotherapy
QA: Quality assurance
LC: Local control
MRI: Magnetic resonance imaging
NSCLC: Non-small-cell lung carcinoma
OMD: Oligometastatic disease
OS: Overall survival
PET: Positron emission tomography
PFS: Progression-free survival
PhT: Photon therapy
PT: Proton therapy
RT: Radiation therapy
RCT: Randomized controlled trials
SBRT: Stereotactic body radiation therapy
